# Apoptosis induced by temozolomide and nimustine in glioblastoma cells is supported by JNK/c-Jun-mediated induction of the BH3-only protein BIM

**DOI:** 10.18632/oncotarget.5274

**Published:** 2015-09-16

**Authors:** Maja T. Tomicic, Ruth Meise, Dorthe Aasland, Nancy Berte, Rebekka Kitzinger, Oliver H. Krämer, Bernd Kaina, Markus Christmann

**Affiliations:** ^1^ Department of Toxicology, University Medical Center Mainz, D-55131 Mainz, Germany

**Keywords:** TMZ, ACNU, high-grade gliomas, BIM, JNK

## Abstract

The outcome of cancer therapy strongly depends on the complex network of cell signaling pathways, including transcription factor activation following drug exposure. Here we assessed whether and how the MAP kinase (MAPK) cascade and its downstream target, the transcription factor AP-1, influence the sensitivity of malignant glioma cells to the anticancer drugs temozolomide (TMZ) and nimustine (ACNU). Both drugs induce apoptosis in glioma cells at late times following treatment. Activation of the MAPK cascade precedes apoptosis, as shown by phosphorylation of Jun kinase (JNK) and c-Jun, a main component of AP-1. Pharmacological inhibition and siRNA mediated knockdown of JNK and c-Jun reduced the level of apoptosis in LN-229 glioma cells treated with TMZ or ACNU. Analyzing the underlying molecular mechanism, we identified the pro-apoptotic gene *BIM* as a critical target of AP-1, which is upregulated following TMZ and ACNU. Importantly, shRNA mediated downregulation of BIM in the malignant glioma cell lines LN-229 and U87MG led to an attenuated cleavage of caspase-9 and, consequently, reduced the level of apoptosis following TMZ and ACNU treatment. Overall, we identified JNK/c-Jun activation and BIM induction as a late pro-apoptotic response of glioma cells treated with alkylating anticancer drugs.

## INTRODUCTION

The therapeutic options for malignant gliomas are still very limited, which is reflected by the low five-year survival of patients (<5%) and the low median patient survival (12–15 months) [1]. Therapy of malignant gliomas is based on the combined use of ionizing radiation and the DNA alkylating agent temozolomide (TMZ) [2]. TMZ exerts its effect *via* induction of *O*^6^-methylguanine (*O*^6^MeG), which represents a mutagenic and cytotoxic lesion [3]. DNA-crosslinking agents such as nimustine (ACNU) and lomustine (CCNU) are used as second-line chemotherapeutics. They chloroethylate the *O*^6^-position of guanine, thereby generating *O*^6^-chloroethylguanine (*O*^6^ClG) [4]. *O*^6^ClG undergoes intramolecular rearrangement to form N1-O^6^-ethenoguanine and finally N1-guanine-N3-cytosine inter-strand crosslinks (ICLs) [5, 6]. Besides high-grade gliomas, *O*^6^-chloroethylating agents are also used to treat malignant melanomas, gastrointestinal and pancreatic cancer, and Hodgkin's and non-Hodgkin's lymphomas [7].

The response to anticancer drug therapy largely depends on the DNA repair activity of the tumor and the activation of the DNA damage response (DDR). Exposure of tumor cells to DNA damaging anticancer drugs causes DNA replication arrest and DNA double-strand breaks (DSB), which are a direct trigger of the DDR. As a consequence, several important transcription factors like p53 and AP-1 become activated [8], which signal either cell survival or death pathways.

Activation of p53 results in cell cycle arrest, which occurs either in the G1 or the G2/M phase [9] and causes the induction of different DNA repair genes [8], thereby triggering resistance to anticancer drugs. On the other hand, p53 can also induce several important pro-apoptotic factors such as the Fas receptor (FAS) [10]. Therefore, the outcome of p53 activation largely depends on the anticancer drug and the cell system used. In malignant glioma cells, p53 provokes resistance to the topoisomerase I inhibitors topotecan and irinotecan [11–14] and the chloroethylating agents ACNU [15] and BCNU [16, 17]. In case of the chloroethylating nitrosoureas, this protective effect is caused by the induction of the nucleotide excision repair genes *xpc* and *ddb2* [15] and the translesion polymerase eta [18]. Unlike chloroethylating agents, p53 stimulates apoptosis in U87MG glioma cells treated with TMZ [19]. Nevertheless, there are also opposite reports showing a protective function of p53 in glioma cells exposed to TMZ [16, 17, 20–22], indicating cell type-specific effects.

A second transcription factor that can be activated following anticancer drug treatment is AP-1, a dimeric transcription factor consisting of proteins belonging to the Fos, Jun or ATF family. AP-1 is activated *via* the MAPK (mitogene-activated protein kinase) pathway, involving JNK (c-Jun N-terminal kinase), p38K (p38 kinase) and ERK1/2 (extracellular signal-regulated kinases 1/2). Upon DNA damage, activation of AP-1 results in the induction of a plethora of AP-1 target genes, including DNA repair genes [8, 23–25] and pro-apoptotic genes [26–29]. Whereas for many genotoxins the activation of the MAPK cascade is experimentally well established [30], it is unclear whether DNA lesions induced by TMZ and CNUs are able to activate the MAPK/p38 kinase and whether this has an impact on therapy. Previously it was reported that JNK inhibition enhances senescence-associated β-galactosidase activity in TMZ-treated glioma cells with functional p53, whereas it induces mitotic catastrophe in p53 mutated cells [31]. Concerning p38K, it was reported that its inhibition sensitizes U87MG cells to TMZ due to abrogation of the G2 arrest [32, 33]. Regarding CNUs, it was reported that knockdown of the AP-1 component FRA1 sensitizes glioma cells towards ACNU *via* the attenuation of CHK1 phosphorylation and abrogation of the G2/M arrest [34], whereas carmustine (BCNU) induced ERK- and JNK-dependent cell death of neuronally-differentiated PC12 cells *via* generation of reactive oxygen species [35].

Here we show for the first time that the MAPK cascade triggered by JNK and its target c-Jun is involved in stimulating apoptosis upon TMZ and ACNU treatment of LN-229 and U87MG glioma cells. The cytotoxic effect results from AP-1 dependent induction of the BH3-only protein BIM, which reveals BIM as an important factor in TMZ and CNU-induced killing of glioma cells.

## RESULTS

### Induction of apoptosis following TMZ and ACNU treatment

**Figure 1:**
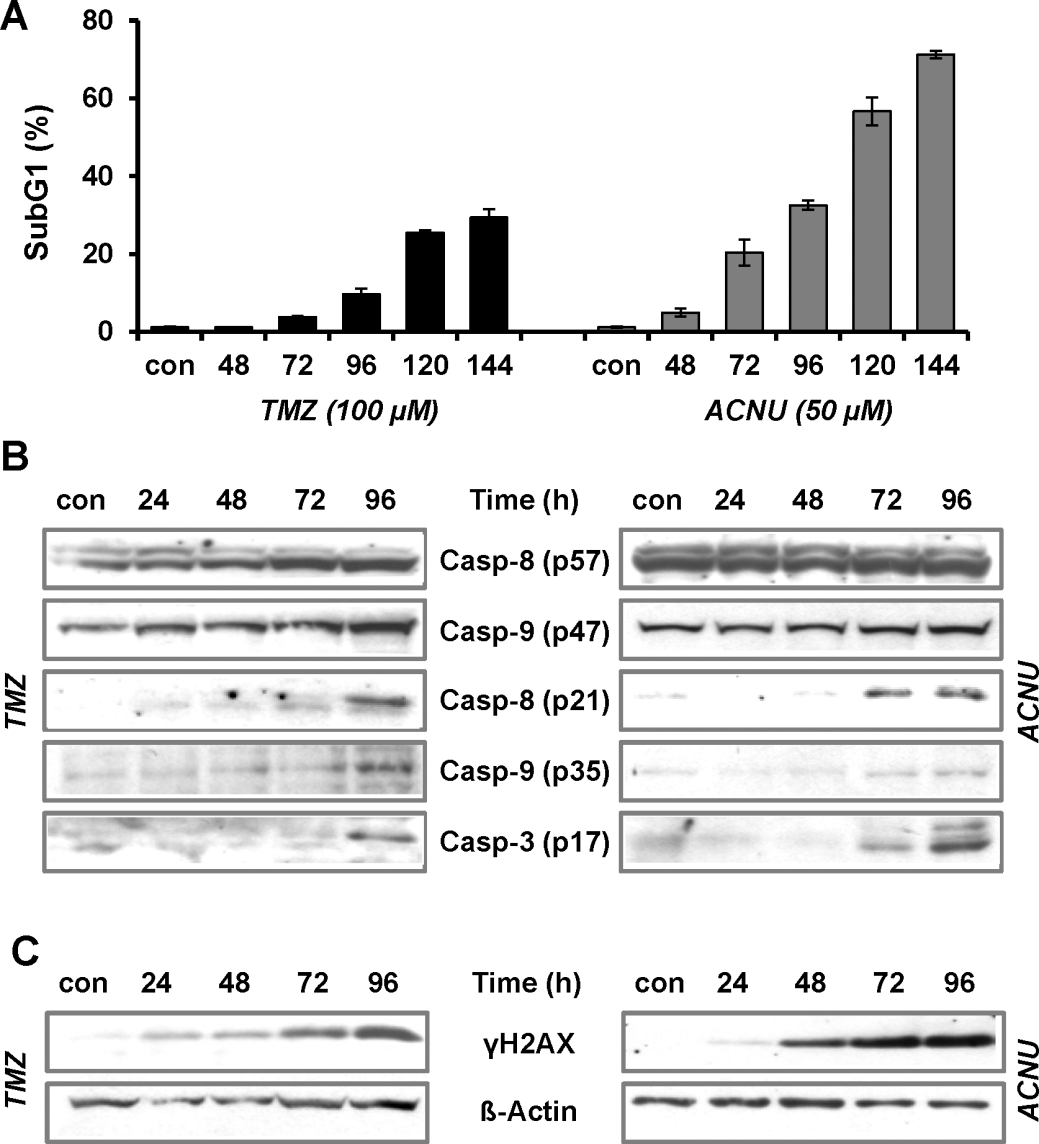
TMZ- and ACNU-induced apoptosis and DNA damage **A.** LN-229 cells were exposed to 100 μM TMZ or 50 μM ACNU. At different time points after exposure cells were stained with PI and the subG1 fraction was determined by flow cytometry. B/C. LN-229 cells were exposed to 100 μM TMZ or 50 μM ACNU for indicated times. Protein extracts were prepared and subjected to western blot analysis. **B.** Expression of procaspase-8 (p57) and -9 (p47) as well as expression of the cleaved caspases-8 (p21), -9 (p35) and -3 (p17) was analyzed using specific antibodies. **C.** Expression of γH2AX was analyzed using specific antibodies. Detection of Δ-Actin was used as loading control.

Exploring the role of AP-1 for the sensitivity of malignant glioma cells to TMZ and ACNU, we first investigated the effectiveness of the anticancer drugs in the induction of apoptosis and the formation of DNA damage. Upon treatment of LN-229 cells with 100 μM TMZ or 50 μM ACNU, concentrations known to be reached in the serum of patients [36], a time-dependent induction of apoptosis was observed (Fig. 1A). Apoptosis started 96 h after TMZ treatment and earlier, after 72 h, in case of ACNU treatment, reaching 25% and 55%, respectively, 120 h after the onset of treatment. Parallel to the induction of cell death, cleavage of caspase-8 and -9 and the effector caspase-3 was observed (Fig. 1B). These events were preceded by phosphorylation of H2AX (γH2AX) (Fig. 1C), indicating activation of the DNA damage response pathway.

### Impact of p53 signaling on TMZ and ACNU-induced cell death and DNA repair in glioma cells

An important transcription factor associated with survival and death is p53. In order to analyze its impact on TMZ and ACNU-induced cytotoxicity, LN-229 glioblastoma cells, wild-type (wt) for p53 (Suppl. Fig. 1A) were pre-treated with the p53 inhibitor pifithrin α (Pthα). This drug enhanced the cytotoxic potential of ACNU, but at the same time slightly protected LN-229 cells against TMZ (Suppl. Fig. 1B, left panel). Moreover we utilized LN-229 pSUPERp53 knockdown cells and LN-229 pSUPER control transfectants [37], which showed similar results (Suppl. Fig. 1B, right panel). Interestingly, despite the different biological outcomes, the expression of proapoptotic (Fas) and prosurvival factors (p21, XPC and DDB2) was similar in p53 wt cells following TMZ and ACNU treatment on mRNA (Suppl. Fig. 1C) and protein level (Suppl. Fig. 1D). The data support the notion that for ACNU the p53-induced activation of NER (induction of XPC and DDB2) is most important for cellular protection [38]. Upon TMZ exposure, activation of NER also occurs. However, it does not contribute to the repair of TMZ-induced DNA damage since O^6^-MeG is repaired via MGMT and not by NER [3]. In case of TMZ, enhanced expression of *Fas* seems to be the predominant p53 regulated pro-apoptotic trait.

### Activation of MAPK signaling upon TMZ and ACNU in glioma cells

**Figure 2:**
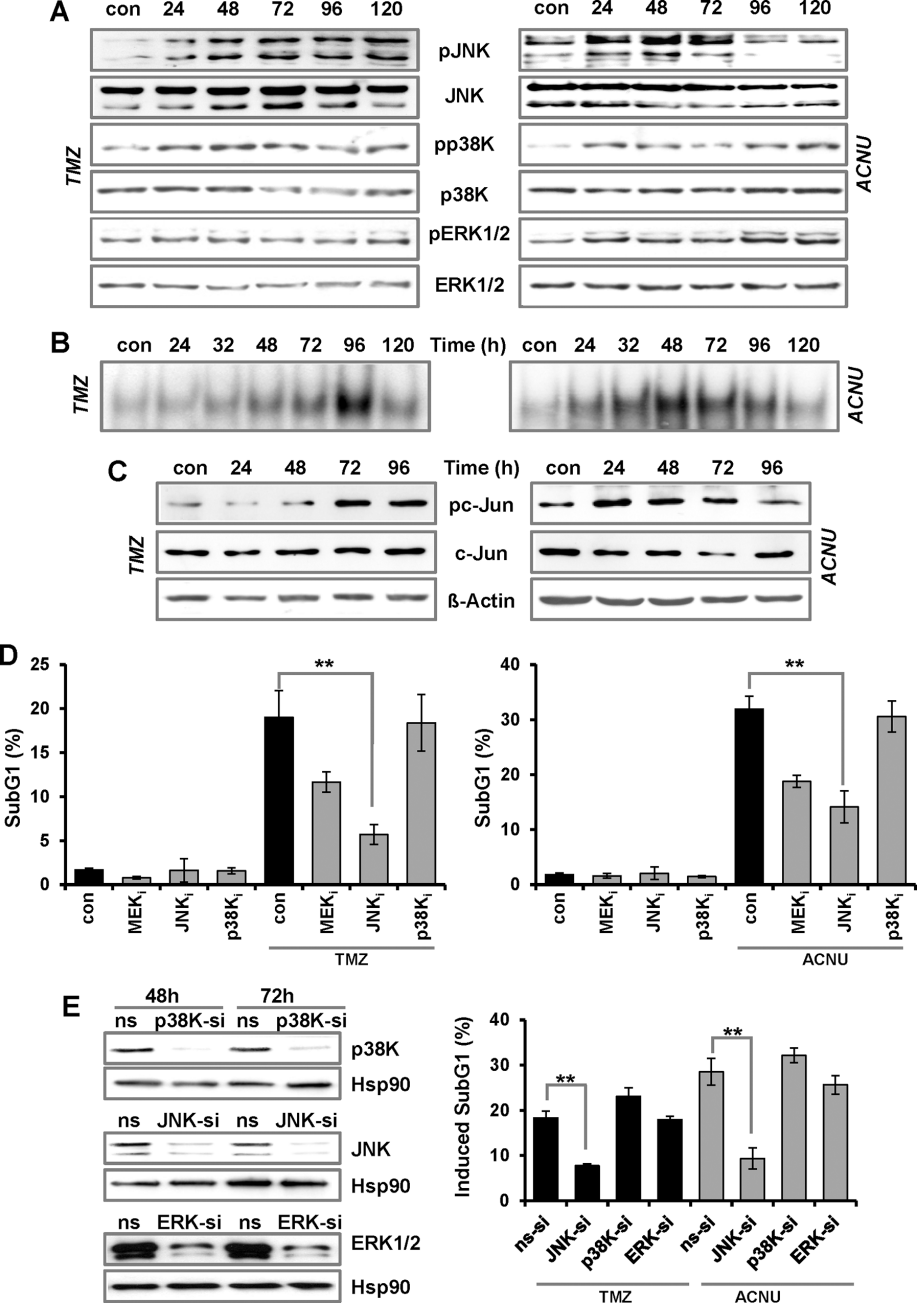
TMZ- and ACNU-induced activation of AP-1 and impact on sensitivity A/B. LN-229 cells were exposed to 100 μM TMZ or 50 μM ACNU for indicated times. Protein extracts were prepared and subjected to western blot analysis. **A.** Expression of JNK, p38K and ERK1/2, as well as expression of the phosphorylated forms (pJNK, pp38K and pERK1/2) was analyzed using specific antibodies. **B.** LN-229 cells were exposed to 100 μM TMZ or 50 μM ACNU for indicated times. Nuclear extracts were isolated and incubated with radioactively labeled oligonucleotides containing the AP-1 binding site of the collagenase promoter (*mmp1)*. The binding of AP-1 was visualized by EMSA. **C.** Expression of c-Jun as well as expression of the phosphorylated form (pc-Jun) was analyzed using specific antibodies. Detection of Δ-Actin was used as loading control. **D.** LN-229 cells were pre-incubated for 1 h with a specific inhibitor for JNK1/2/3 (SP600125), p38K (SB203580) and MEK1/2 (U0126) thereafter not exposed or exposed to 100 μM TMZ for 120 h or to 50 μM ACNU for 96 h. Cells were stained with PI and the subG1 fraction was determined by flow cytometry. **E.** LN-229 cells were transfected with siRNA against JNK, p38K and ERK1/2 or a non-silencing RNA (ns). Expression of JNK, p38K and ERK1/2 was analyzed 48 and 72 h later using specific antibodies. Detection of Hsp90 was used as loading control (left panel). LN-229 cells were exposed or not exposed to 100 μM TMZ or 50 μM ACNU. 24 h after exposure, cells were transfected with 50 nM siRNA against JNK, p38K and ERK1/2 or a non-silencing RNA (ns). Apoptosis was measured 120 h upon TMZ and 96 h after the onset of ACNU treatment *via* measurement of the subG1 fraction (right panel). Apoptotic frequency in relation to the untreated (siRNA transfection) control is shown.

In addition to p53, the MAPK cascade also becomes activated upon treatment of glioma cells with TMZ and ACNU. Thus, TMZ induced a long-lasting phosphorylation of the p38K and the JNK starting 24 h and the reaching its maximum 72–120 h after the begin of treatment (Fig. 2A, left panel). Both kinases became strongly phosphorylated 24–72 h after ACNU treatment, thereafter the phosphorylation of JNK declined (Fig. 2A, right panel). ACNU, but not TMZ, enhanced the phosphorylation of the ERK kinase (ERK1/2). In effect of this, downstream transcription factors were activated as shown by increased AP-1 binding activity (Fig. 2B) and phosphorylation of its main component c-Jun (Fig. 2C).

### Impact of the MAPK cascade on TMZ and ACNU-induced cell death in glioma cells

To analyze whether activation of the MAPK cascade has an impact on the sensitivity to TMZ and ACNU, the kinases were inhibited and the drug sensitivity was analyzed by determining the apoptosis frequency. Inhibition of JNK by a JNK1/2/3 inhibitor (SP600125) clearly protected LN-229 cells against TMZ and ACNU (Fig. 2D). Inhibition of p38K (SB203580) had no effect on cell death whereas inhibition of ERK1/2 by U0126 (an inhibitor of the upstream MEK1/2 kinase) slightly protected the cells (Fig. 2D). To further analyze the impact of the MAPK cascade on the sensitivity to TMZ and ACNU, we repressed JNK, p38K and ERK1/2 with specific siRNAs (Fig. 2E, left panel). Also in this case, knockdown of JNK strongly protected the cells against TMZ and ACNU (Fig. 2E, right panel). On the other hand, knockdown of ERK1/2 and p38K had no effect on TMZ/ACNU-induced apoptosis.

**Figure 3:**
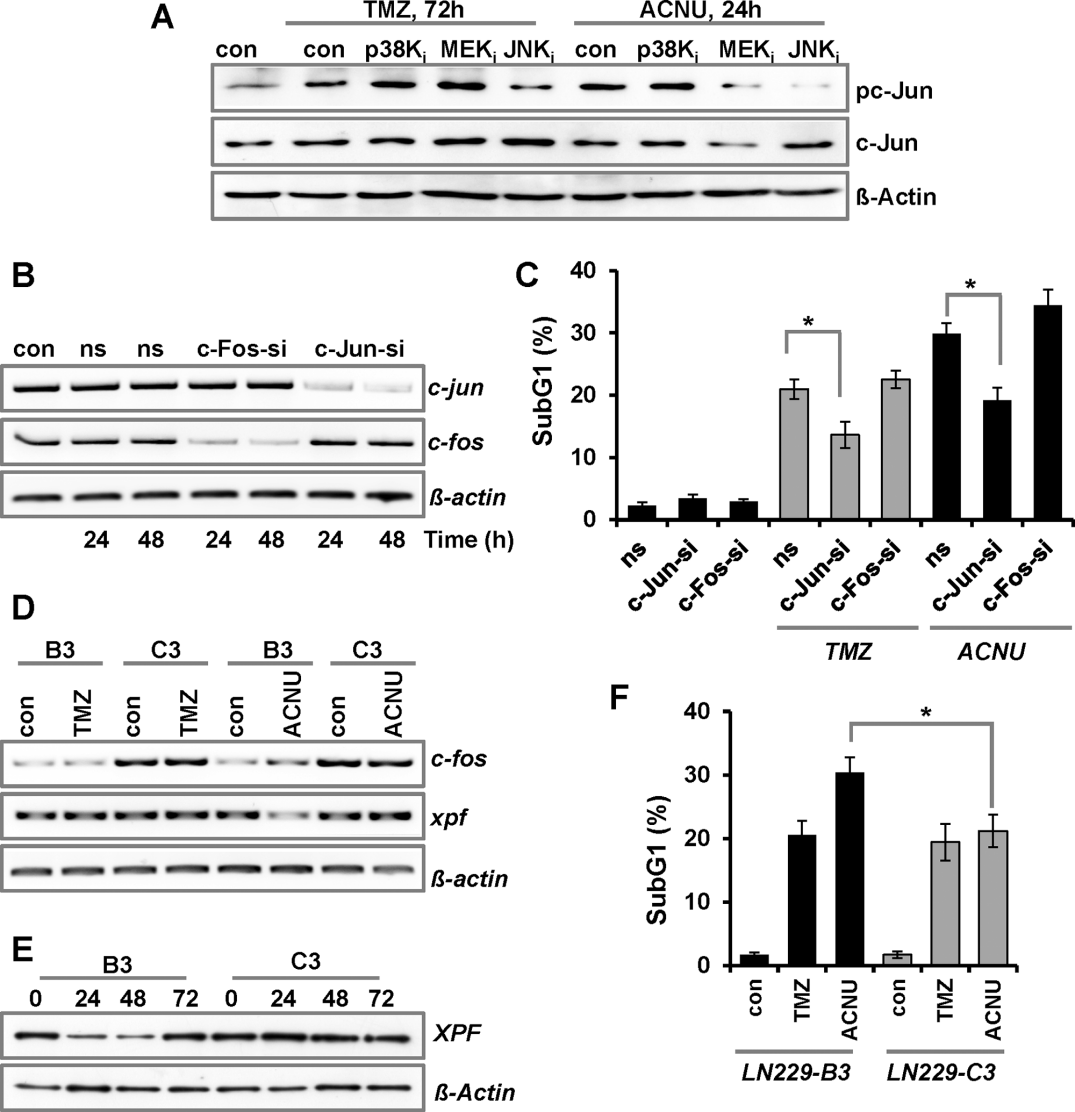
Impact of c-Jun and c-Fos on TMZ- and ACNU-induced cell death **A.** LN-229 cells were pre-incubated for 1 h with a specific inhibitor for JNK1/2/3 (SP600125), p38K (SB203580) or MEK1/2 (U0126) and thereafter not exposed or exposed to 100 μM TMZ for 72 h or to 50 μM ACNU for 24 h. Expression of c-Jun and its phosphorylated form (pc-Jun) was analyzed using specific antibodies. Detection of Δ-Actin was used as loading control. B/C. LN-229 cells were transfected with 20 nM siRNA directed against c-Jun (si-cJun) and c-Fos (si-cFos) or a non-silencing RNA (ns). **B.** 24 and 48 h later RNA was isolated and endpoint RT-PCR was performed using *c-fos, c-jun* or, as loading control, *gapdh* specific primers. **C.** 24 h later cells were not exposed or exposed to 100 μM TMZ for 120 h or 50 μM ACNU for 96 h, stained with PI and the sub-G1 fraction was determined by flow cytometry. **D–F.** LN-229 cells stably overexpressing c-Fos (C3) and LN-229 mock-transfected cells (B3) were exposed to 100 μM TMZ or 50 μM ACNU. D. 24 h later RNA was isolated and endpoint RT-PCR was performed using *c-fos, xpf* or, as loading control, *gapdh* specific primers. E. At different time points upon ACNU exposure, expression of XPF was analyzed. **F.** 96 h after exposure to ACNU cells were stained with PI and the subG1 fraction was determined by flow cytometry.

Next, we analyzed which kinase is responsible for the activation of AP-1 after treatment with TMZ and ACNU. Therefore, phosphorylation of the main AP-1 component c-Jun was analyzed in cells pre-treated with the kinase inhibitors. The results indicate that JNK, but not p38K or ERK1/2 activation, is responsible for c-Jun phosphorylation upon TMZ and ACNU (Fig. 3A). To analyze whether the pro-apoptotic effect of JNK activation is caused by c-Jun, siRNA-mediated knockdown was used to abrogate the expression of c-Jun or another component of the AP-1 complex, c-Fos (Fig. 3B). Knockdown of c-Jun, but not c-Fos, provoked an effect comparable to the JNK inhibitor, namely protection against TMZ and ACNU-induced cytotoxicity (Fig. 3C), indicating that the pro-apoptotic effect triggered by the MAPK cascade activation is caused by c-Jun. In contrast, knockdown of c-Fos did not protect against TMZ-induced apoptosis and even slightly sensitized cells to ACNU (Fig. 3C).

This sensitization effect deserves to be considered in more detail. Previously, a protective function of c-Fos was reported in human fibroblasts treated with UV-light, which was associated with the induction and re-synthesis of the NER-endonuclease XPF [24, 25]. In LN-229 cells, 24 h after exposure to ACNU, both *c-fos* and *xpf* were slightly reduced; however, at later times *c-fos* became induced and the basal expression of *xpf* was restored (Suppl. Fig. 2A). To analyze whether c-Fos is responsible for the re-synthesis of *xpf*, we utilized LN-229 cells overexpressing c-Fos (Suppl. Fig. 2B, clone C3). Indeed c-Fos overexpressing cells showed no reduction of *xpf* mRNA following ACNU treatment, whereas reduction still occurred in the mock-transfected clone B3 (Fig. 3D). An accumulation of XPF was also observed at protein level (Fig. 3E), which was associated with a reduced sensitivity to ACNU (Fig. 3F). These data corroborate a protective function of c-Fos against ACNU-induced cell death by stimulation of NER. Overall, the data indicate that in LN-229 cells treated with TMZ or ACNU, MAPK activation leads to enhanced cell death which is regulated by JNK and c-Jun, but not c-Fos. For ACNU, this process appears to be counteracted by c-Fos triggered stimulation of XPF dependent repair of chloroethylated DNA adducts.

### MAPK cascade and the expression of proapoptotic factors following TMZ and ACNU exposure

**Figure 4:**
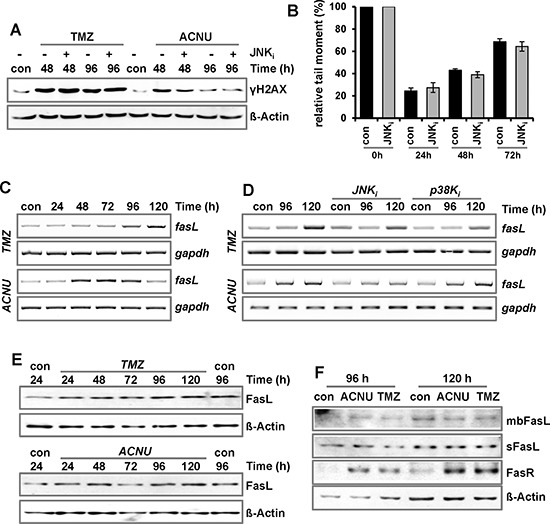
Impact of MAPK-signaling on DNA repair and expression of FasL A/B. LN-229 cells were pre-incubated for 1 h with a specific inhibitor for JNK1/2/3 (SP600125) and thereafter non-exposed or exposed to 100 μM TMZ or 50 μM ACNU for 24 h. **A.** At indicated time points expression of γH2AX was analyzed using specific antibodies. Detection of Δ-Actin was used as loading control. **B.** Cells were non-exposed or exposed to 50 μM ACNU. At indicated time points, cells were harvested and subsequently irradiated with 8 Gy. Thereafter, the alkaline comet assay was performed. **C.** LN-229 cells were exposed to 100 μM TMZ (upper panel) or 50 μM ACNU (lower panel). **D.** LN-229 cells were pre-incubated for 1 h with a specific inhibitor for JNK1/2/3 (SP600125) or p38K (SB203580) and thereafter non-exposed or exposed to 100 μM TMZ (upper panel) or 50 μM ACNU (lower panel). **C/D.** At indicated time points RNA was isolated and endpoint RT-PCR was performed using *fasL* or, as loading control, *gapdh* specific primers. **E/F.** LN-229 cells were exposed to 100 μM TMZ or 50 μM ACNU. **E.** At indicated time points expression of the FasL was analyzed. **F.** 96 and 120 h later the expression of the soluble FasL (sFasL), the membrane bound form of the FasL (mbFasL) and the FasR was analyzed.

To analyze whether activation of JNK/c-Jun negatively influences DNA damage signaling and repair, the TMZ/ACNU-induced phosphorylation of H2AX (γH2AX) was analyzed upon inhibition of JNK (Fig. 4A). Furthermore, in the case of ACNU, the formation and repair of interstrand crosslinks was analyzed (Fig. 4B). In none of the experiments, inhibition of JNK had an effect, indicating that JNK has no negative impact on the cell's DNA damage signaling and repair capacity. Therefore, we posit that the JNK/c-Jun axis enhances glioma cell sensitivity via stimulation of the apoptotic pathway. An important AP-1 induced proapoptotic gene encodes the Fas ligand, a factor of the extrinsic apoptotic pathway [28, 39]. Indeed, treatment of LN-229 cells with TMZ and ACNU led to an enhanced expression of *fasL* mRNA (Fig. 4C). In the case of TMZ, *fasL* induction was slightly reduced by inhibition of JNK and p38K, and in the case of ACNU, JNK inhibition completely abrogated *fasL* induction whereas p38K inhibition had no effect (Fig. 4D). This indicates that in TMZ- and ACNU-treated glioma cells the *fasL* gene is a target of AP-1. However, in contrast to *fasL* mRNA, only a weak induction of the FasL protein (Fig. 4E) and no translocation of it to the outer cell membrane were observed (Fig. 4F). Thus, we conclude that the *fasL* is most likely not responsible for the pro-apoptotic effect of JNK/c-Jun.

**Figure 5:**
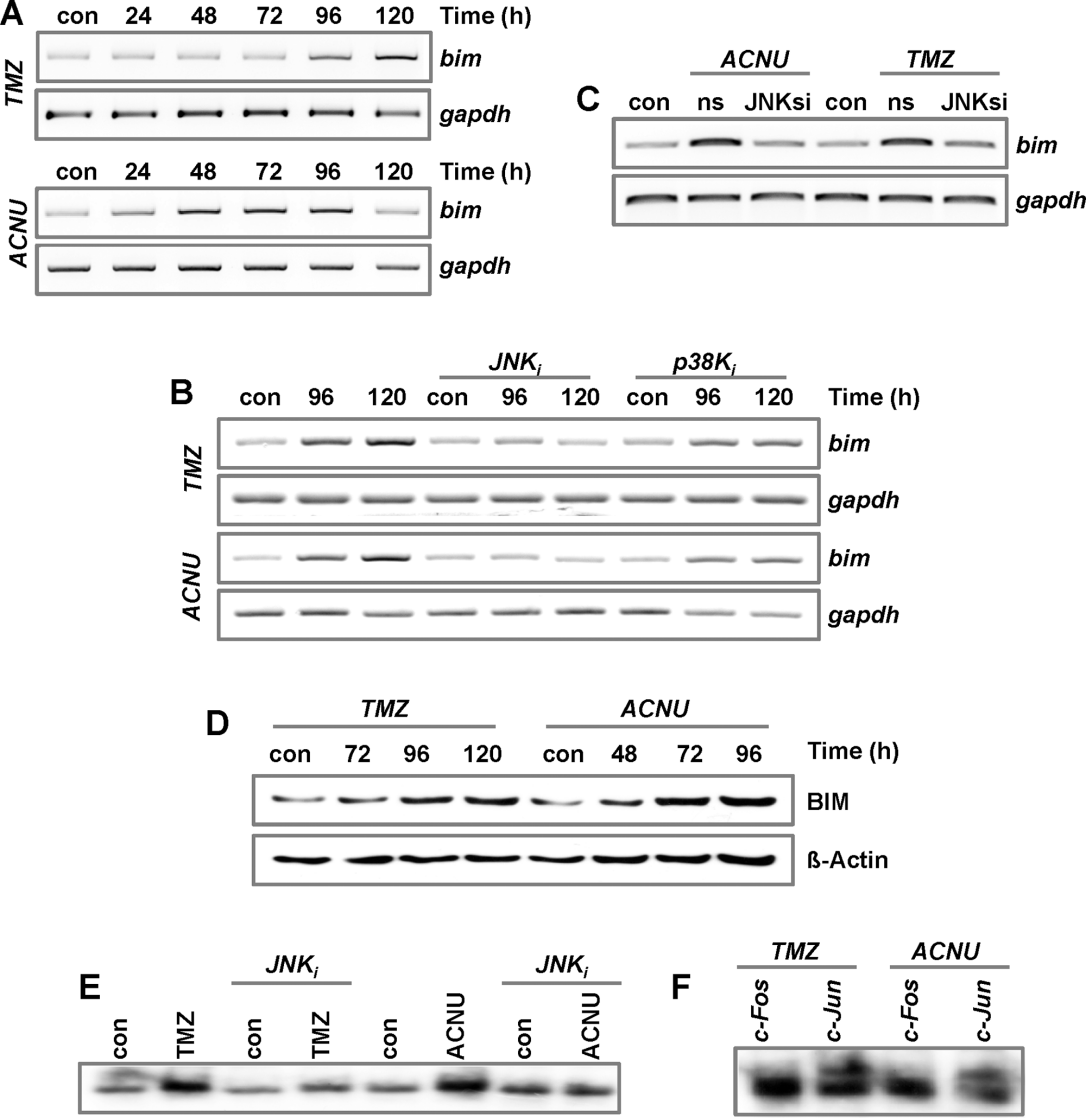
Impact of MAPK-signaling on the expression of BIM **A.** LN-229 cells were exposed to 100 μM TMZ or 50 μM ACNU. **B.** LN-229 cells were pre-incubated for 1 h with a specific inhibitor for JNK1/2/3 (SP600125) or p38K (SB203580) and thereafter non-exposed or exposed to 100 μM TMZ (upper panel) or 50 μM ACNU (lower panel). **C.** LN-229 cells were exposed to 100 μM TMZ or 50 μM ACNU. 24 h later, cells were transfected with 50 nM siRNA against JNK or a non-silencing control RNA (ns). A-C. At indicated time points RNA was isolated and endpoint RT-PCR was performed using *bim* or, as loading control, *gapdh* specific primers. **D.** LN-229 cells were exposed to 100 μM TMZ or 50 μM ACNU and expression of BIM was analyzed at indicated time points. **E.** LN-229 cells were pre-incubated for 1 h with a specific inhibitor for JNK1/2/3 (SP600125) or p38K (SB203580) and thereafter non-exposed or exposed to 100 μM TMZ (for 120 h) or 50 μM ACNU (for 96 h). Nuclear extracts were isolated and incubated with radioactively labeled oligonucleotides containing the AP-1 binding site of the *bim* promoter. The binding of AP-1 was visualized by EMSA. **F.** EMSA supershift assay: The binding of c-Jun and c-Fos to oligonucleotides containing the AP-1 binding site of the *bim* promoter was analyzed by the addition of specific antibodies to the EMSA reaction utilizing proteins from TMZ- and ACNU-exposed cells.

AP-1 was also shown to induce *cyclinA, cyclinD1 and p16^INK4^* [40–43], which are involved in cell cycle regulation, and the proapoptotic genes *bak* and *bim* [26, 27]. Upon TMZ and ACNU we observed in LN-229 cells only the induction of *bim* (Fig. 5A, Suppl. Fig. 2C and data not shown). For *BIM*, three proapoptotic isoforms (*bim_EL_*, *bim_L_*, and *bim_S_*) were described. In our experiments, induction of *bim_EL_* was observed (Suppl. Fig. 2D). Similar to *fasL*, induction of *bim_EL_* in TMZ- and ACNU-treated cells was abrogated by pharmacological inhibition of JNK, but not p38K (Fig. 5B). The importance of JNK for the transcriptional activation of BIM was further shown by using JNK siRNA, leading to abrogation of BIM induction (Fig. 5C). Most importantly, BIM was also induced on protein level (Fig. 5D).

To further verify that *BIM* is an AP-1 target in glioma cells, EMSA assays were performed using radioactively labeled oligonucleotides harboring the potential AP-1 binding site of the *BIM* promoter (Fig. 5E). These experiments, together with supershift experiments, demonstrate the binding of c-Jun, but not of c-Fos, to this AP-1 binding site in cell extracts of TMZ- and ACNU-treated LN-229 cells (Fig. 5F).

### BIM stimulates TMZ- and ACNU-induced death in glioma cells

**Figure 6:**
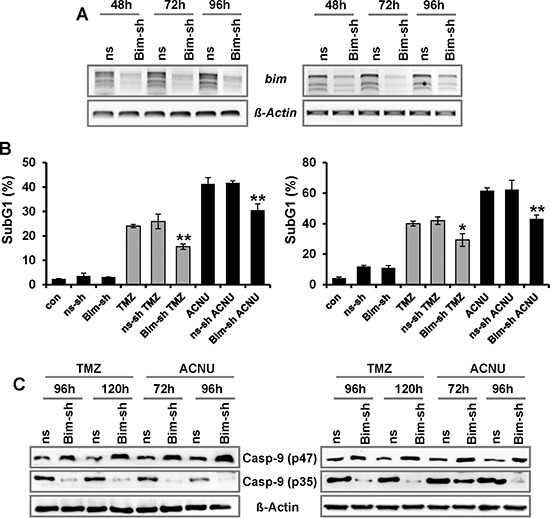
Impact of BIM on TMZ- and ACNU-induced cell death **A–C.** LN-229 (left panel) and U87MG (right panel) glioblastoma cells were transiently transfected with plasmids expressing two different shRNA constructs specific for BIM (BIM-sh) or plasmids expressing a non-silencing shRNA (ns-sh). **A.** At indicated time points RNA was isolated and endpoint RT-PCR was performed using *bim* or, as loading control, *Δ-actin* specific primers. **B.** 24 h later cells were non-exposed or exposed to 100 μM TMZ (for 120 h) or 50 μM ACNU (for 96 h). Cells were stained with PI and the sub-G1 fraction was determined by flow cytometry. **C.** 24 h later cells were exposed to 100 μM TMZ or 50 μM ACNU and 120 or 96 later the expression of procaspase-9 (p47) as well as the expression of cleaved caspases-9 (p35) was analyzed using specific antibodies.

To analyze whether BIM has an impact on the resistance of glioblastoma cells to TMZ and ACNU, BIM was transiently down-regulated by shRNAs in LN-229 and U87MG cells. Expression of *BIM* was determined by PCR, showing a strongly reduced expression 48 to 96 h after transfection (Fig. 6A, the effect on all three isoforms is shown). The response of mock-transfected and BIM-downregulated glioma cells to TMZ and ACNU was determined by flow cytometry. Apoptosis occurred at a significantly lower frequency in cells transfected with BIM-shRNA plasmid than in mock-transfected cells (Fig. 6B). Furthermore, cleavage of caspase-9 was significantly reduced upon knockdown of BIM (Fig. 6C). The data demonstrate a direct involvement of BIM in the induction of the caspase-9 driven apoptotic pathway in glioma cells following TMZ and ACNU treatment.

## DISCUSSION

Here, we analyzed the impact of the MAPK cascade and its target, the transcription factor AP-1, on the sensitivity of glioma cells to TMZ and ACNU, which are important alkylating drugs used in glioma therapy.

TMZ and ACNU induce apoptosis at late times in glioma cells (this data and [15]). The activation of the MAPK cascade precedes the induction of apoptosis, as shown by phosphorylation of p38K and JNK, increased phosphorylation of c-Jun and enhanced AP-1 binding activity. Inhibition and siRNA mediated knockdown of JNK protected LN-229 cells against TMZ- and ACNU-induced apoptosis. The same result was obtained upon siRNA-mediated silencing of the AP-1 component c-Jun, but not of c-Fos, indicating that the proapoptotic effect of JNK activation is caused by c-Jun. Interestingly, knockdown of c-Fos slightly sensitized LN-229 cells to ACNU, which was explained by lack of upregulation of the NER protein XPF.

We are aware that our data reporting JNK inhibition to block apoptosis in TMZ-treated cells are in contrast to Ohba *et al*., showing that inhibition of JNK enhances the TMZ-induced cytotoxicity in U87MG cells [31]. In these experiments cell death was analyzed by the MTT assay 72 h after TMZ exposure. In our hands, apoptosis is not yet executed in LN-229 or U87MG cells at this time following treatment (Suppl. Fig. 3A). Using the MTT assay, we also observed a reduced metabolic activity in U87MG cells upon JNK inhibition, whereas in LN-229 cells this effect was only marginal (Suppl. Fig. 3B). Further, Ohba *et al*. reported that the increased sensitivity following JNK inhibition is associated with enhanced senescence while the effect on apoptosis was not analyzed. Taken together, it is reasonable to conclude that JNK signals different events, depending on the time period following exposure. In line with this, we previously showed that glioma cells undergo autophagy, senescence and apoptosis in a specific time-dependent manner after TMZ treatment [44]. In this case, autophagy, which is the earliest event upon TMZ treatment, stimulates cells to undergo senescence rather than apoptosis, showing that the different endpoints are interrelated. Furthermore, inhibition of autophagy and thereby senescence leads to an increase in apoptosis upon TMZ [44], explaining why inhibition of JNK at the same time can enhance senescence [31] and reduce apoptosis (this paper). In another study, sensitization of glioma cells to TMZ by JNK inhibitors was observed in MGMT expressing cells due to an impact of JNK on the basal expression of this DNA repair gene [45]. Both cell lines used in our study (LN-229 and U87MG) are MGMT deficient (due to promoter methylation) and, therefore, a possible repression of MGMT following JNK inhibition cannot explain the data reported here.

To investigate the molecular mechanism underlying JNK/c-Jun-induced apoptosis in TMZ- and ACNU-treated glioma cells, we analyzed whether JNK/c-Jun is directly involved in the activation of the extrinsic apoptotic pathway. The extrinsic pathway is activated *via* binding of FasL to the Fas receptor (Fas) [46, 47]. While the FasR is induced *via* p53, the FasL is a prominent target of AP-1 [28, 29, 39]. Previously, we showed that apoptosis induced by UV-light and cisplatin is triggered by JNK/c-Jun mediated induction of FasL [48, 49]. Here, we demonstrate that LN-229 cells treated with TMZ and ACNU respond with an enhanced expression of *fasL* mRNA, which was abrogated by inhibition of JNK. However, only weak induction on the protein level and no translocation to the cell membrane was observed, indicating that the transcriptional induction of *fasL* alone is not responsible for the proapoptotic activity of JNK/c-Jun following TMZ and ACNU exposure.

A search for additional AP-1 induced apoptotic targets revealed the induction of the proapoptotic *BIM* gene upon TMZ/ACNU exposure. BIM was originally identified as a Bcl-2-interacting protein [50]. Multiple isoforms were characterized, however only for Bim_EL_, Bim_L_, and BIM_S_ the pro-apoptotic function has been confirmed, BIM_S_ being the most potent. This is partly explained *via* the sequestration of BIM_EL_ and BIM_L_ by the cytoskeleton-associated motor complex and binding to dynein light chain LC8, from which they are released upon various apoptotic stimuli [51]. In our experiments, we observed strong induction of *bim_EL_* (Suppl. Fig. 2C). BIM induces apoptosis by causing mitochondrial permeabilization and the release of apoptogenic factors (e.g. cytochrome c, Smac/DIABLO). In this process, BIM can bind BAX and BAK and induces cytochrome c release from mitochondria [52]. In addition, it was reported that BIM preferentially activates BAX whereas BAK is mostly activated by BID [53]. Upon formation, these factors can activate caspases, including caspase-9, and thereby induce apoptosis [54].

The impact of JNK/c-Jun on the upregulation of *BIM* was shown by the finding that *BIM* induction following TMZ and ACNU was abrogated by pharmacological inhibition and siRNA based silencing of JNK. Also, binding of AP-1/c-Jun to the AP-1 binding site of the *BIM* promoter was demonstrated by EMSA experiments. The data are in line with reports showing AP-1 dependent induction of *BIM* following exposure to nitric acid and upon nerve growth factor deprivation [26, 27]. Moreover, *BIM* was shown to be upregulated in U87MG cells treated with lovastatin [55]. Interestingly, BIM induction was also observed in head and neck squamous cell carcinoma (HNSCC) cell lines upon replication stress provoked by the ribonucleotide reductase inhibitor hydroxyurea (HU) [56]. Since ACNU and TMZ induce DNA replication blocking lesions and DSBs in the 1^st^ and 2^nd^ post-exposure cell cycle, respectively, we suppose that these lesions represent the initial trigger for BIM induction [57, 58].

Besides transcriptional induction, BIM activity can also be regulated via post-translational modifications. Thus ERK1/2-dependent phosphorylation of Bim_EL_ promotes its dissociation from Mcl-1 and Bcl-x and causes its proteasomal degradation [59, 60]. Therefore we analyzed the expression of BIM upon siRNA mediated knockdown of ERK1/2. The results show that knockdown of ERK1/2 leads to an increased expression of BIM, supporting a role of ERK1/2 in controlling BIM stability in glioma cells (Suppl. Fig. 3C). However, ERK1/2 induced degradation of BIM seems not to contribute to TMZ/ACNU sensitivity, as indicated by ERK1/2 knockdown experiments (Fig. 2E). We should note that pharmacological inhibition of MEK1/2, but not siRNA based repression of ERK1/2, slightly protected glioma cells against TMZ and ACNU. This may indicate that the effect of MEK1/2 inhibition may be caused either by ERK1/2-independent signals activated by MEK1/2 or by off-target effects of the inhibitor.

To address the question of whether BIM is involved in TMZ/ACNU-induced apoptosis in glioma cells, experiments were performed using shRNA-mediated silencing of BIM. These experiments showed that knockdown of BIM reduced the cleavage of caspase-9 and the level of apoptosis in LN-229 and U87MG cells treated with TMZ or ACNU. Our findings are in line with a report showing that the JNK activator anisomycin can sensitize glioma cells to apoptosis in a mechanism requiring both JNK activation and BIM upregulation [61]. Furthermore it was shown that the sphingosine analogue FTY720, which also induces upregulation of BIM, synergistically induces apoptosis in combination with TMZ in brain tumor stem cells [62]. A synergistic effect of combined anisomycin/TMZ treatment was also observed in our study (Suppl. Fig. 3D). Therefore, it is reasonable to conclude that targeting BIM represents a novel concept in anticancer therapy [63].

In summary, we show that TMZ and ACNU induce the activation of JNK and subsequently of c-Jun/AP-1 in glioma cells. Pharmacological inhibition and siRNA mediated repression of JNK and c-Jun reduced the level of apoptosis in these cells treated with TMZ and ACNU. Apoptosis was a result of AP-1 triggered induction of the proapoptotic protein BIM, indicating that BIM represents an important factor that contributes to the outcome of TMZ- and ACNU-based therapy of malignant gliomas.

## MATERIALS AND METHODS

### Cell lines and anticancer drug treatment

The glioma cell lines LN-229 and U87MG were described previously [37]. The glioma cell lines LN229-Super and LN229-p53sh were kindly provided by Prof. Weller (Laboratory of Molecular Neuro-Oncology, University Hospital and University of Zurich, Zurich, Switzerland). The cells were grown in Dulbecco's minimal essential medium (DMEM) containing 10% fetal bovine serum (FBS), in 7% CO2 at 37°C. The JNK1/2/3 inhibitor SP600125 and the p38K inhibitor SB203580 were purchased from Sigma-Aldrich (Hamburg, Germany); the MEK1/2 inhibitor U0126 was from Promega (Mannheim, Germany). The inhibitors were added to the medium at a concentration of 10 μM 1 h prior to ACNU or TMZ treatment and were not removed until harvest.

### Knockdown experiments

Knockdown of c-Fos and c-Jun was performed using 20 nM siRNAs from Santa Cruz (sc29221 and sc29223). Knockdown of JNK, p38K and ERK1/2 was performed using 50 nM SignalSilence^®^ SAPK/JNK siRNA I #6233, SignalSilence^®^ p38 MAPK siRNA I #6564, and SignalSilence^®^ p44/42 MAPK (Erk1/2) siRNA #6560. For transfection the Lipofectamine RNAiMAX Transfection Kit (Invitrogen) was used. Down-regulation of BIM was achieved by the transfection with shRNA vectors [56].

### Preparation of cell extracts and western blot analysis

Whole cell extracts and nuclear cell extracts were prepared as described [25]. Proteins were separated by SDS-PAGE and transferred onto a nitrocellulose membrane (Amersham) by semi-dry blotting. For immuno-detection, the antibodies against Δ-Actin (C4, Santa Cruz), BIM (B7929, Sigma), FasL (G247-4, BD Pharmingen), ERK2 (sc-154, Santa Cruz), γH2AX (#05-164, Upstate), XPF (Ab-5, NeoMarkers), c-Fos (sc-52, Santa Cruz) were diluted 1:1000 in 5% non-fat dry milk/Tween-TBS. For western blot analysis with phospho-specific antibodies, cells were directly lyzed in 1 x SDS-PAGE sample buffer (Roti^®^-Load 1, Carl Roth GmbH) and subsequently sonified. Rabbit phospho-specific antibodies (anti-p-cJun: #3270, anti-p-JNK: #4668P; anti-p-p38K: #4511P; anti-p-ERK1/2: #4370P) as well as non- phospho-specific antibodies (anti-cJun: #9165; anti-JNK: #9258P; anti-p38K: #9212P and anti-ERK1/2: #4695P) were purchased from Cell Signaling Technology (Boston, MA, USA) and diluted 1:1000 in 5% BSA/Tween-TBS. The secondary anti-rabbit or anti-mouse antibodies (Rockland) were diluted 1:2000 in 5% non-fat dry milk/Tween-TBS.

### Preparation of RNA and endpoint PCR

Total RNA was isolated using the RNA II Isolation Kit (Machery and Nagel, Düren, Germany) and cDNA synthesis was performed using the Verso cDNA Kit (Thermo Scientific, Bonn, Germany). Endpoint PCR was performed using Red-Taq Ready Mix (Sigma-Aldrich). The specific primers are shown in Suppl. Table 1.

### Determination of apoptosis

For monitoring drug-induced apoptosis and cell cycle distribution, ethanol-fixed cells were treated with DNAse free RNAse and stained with propidium iodide (PI). The Sub-G1 fraction was determined by flow cytometry. The experiments were repeated at least three times. Mean values ± SD are shown.

### Preparation of nuclear extracts and EMSA

Nuclear cell extracts were prepared and subjected to electromobility shift assay (EMSA) or Western blot analysis as described [64]. The sequence of the oligonucleotides specific for the AP-1 binding site of the *mmp1* promoter was 5′-*AGTGGTGACTCATCACT*-3′. The sequence of the oligonucleotides specific for the AP-1 binding site of the *BIM* promoter was 5′-*GTTAGCGGTGACTCACATTCCCAG*-3′. For supershift analysis, 2 μg antibodies specific for c-Jun (sc45, Santa Cruz) or c-Fos (sc-52, Santa Cruz) were incubated with 8 μg protein extract for 20 min at room temperature in the reaction buffer before addition of the labeled oligonucleotides.

### ICL repair measured by single cell gel electrophoresis (SCGE, comet assay)

For the modified comet assay [65] cells were treated with 50 μM ACNU and, after the indicated time periods, cells were trypsinized and washed with ice cold PBS. Thereafter, all cells were irradiated by 8 Gy. Immediately after irradiation, alkaline cell lysis and electrophoresis was performed as described [66]. The results are expressed as relative tail moment (%) = fluorescence intensity tail (%) x distance head center to tail end.

## SUPPLEMENTARY FIGURES AND TABLE



## Abbreviations

AP-1: (activator protein 1)
JNK: (c-Jun N-terminal kinase)
DSB: (DNA double-strand breaks)
ACNU: (nimustine, (1-(4-amino-2-methyl-5-pyrimidinyl) methyl-3-(2-chloroethyl)-3-nitrosourea hydrochloride)
TMZ: (temozolomide)
