# Supplementary material for: Apoptosis induced by temozolomide and nimustine in glioblastoma cells is supported by JNK/c-Jun-mediated induction of the BH3-only protein BIM
Source: Oncotarget. 2015 Sep 16;6(32):33755–68. doi: 10.18632/oncotarget.5274 (PMC4741800; doi:10.18632/oncotarget.5274)
Supplement: Supplementary file 1 [file oncotarget-06-33755-s001.pdf]

SUPPLEMENTARY FIGURES AND TABLE

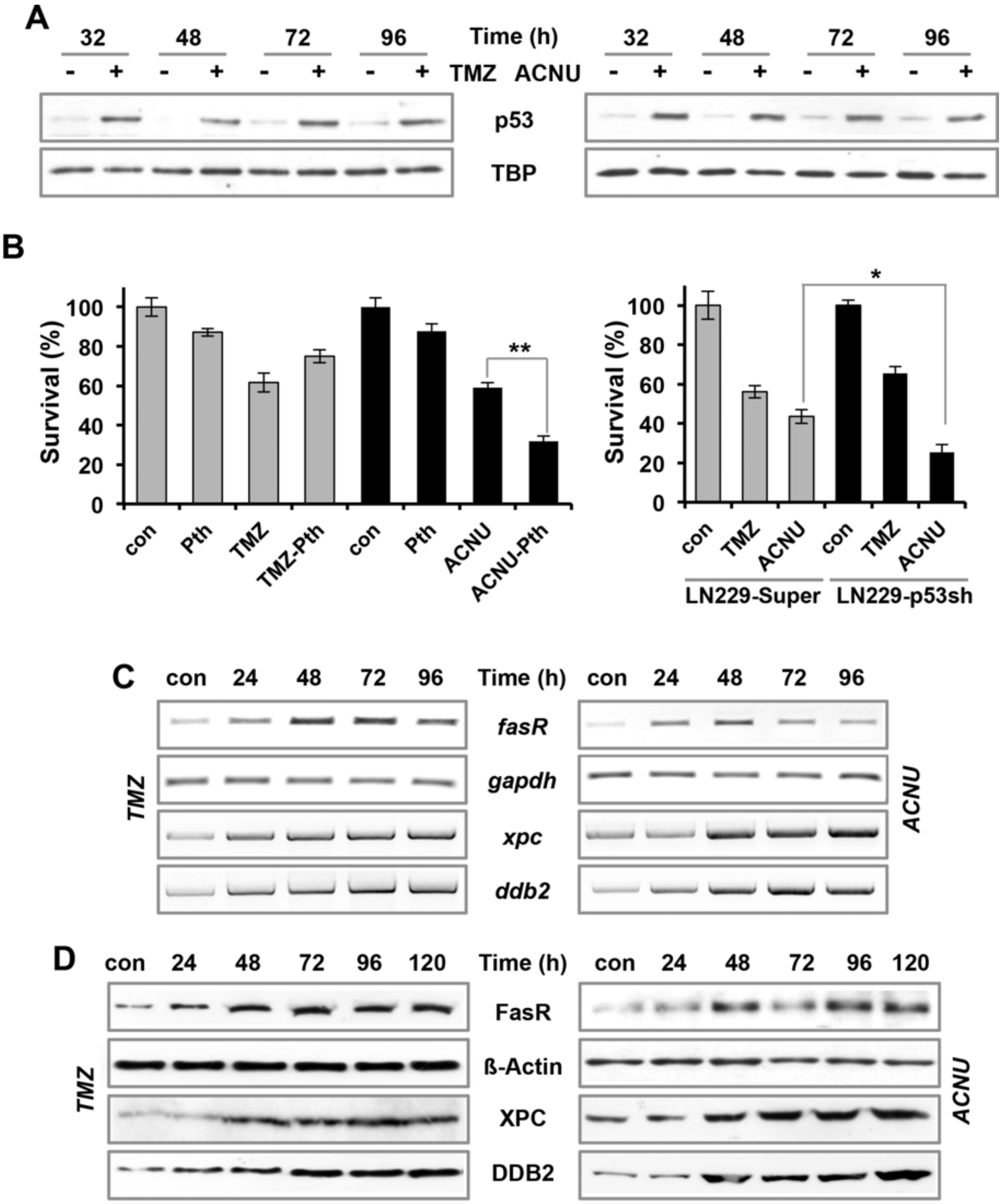

Supplementary Figure S1: TMZ- and ACNU-induced activation of p53 and impact on sensitivity. A. LN-229 cells were exposed to 100 µM TMZ or 50 µM ACNU for the indicated times. Nuclear extracts were isolated, subjected to western blot analysis and expression of p53 was analyzed. Detection of TBP was used as loading control. **B.** LN-229 cells were pre-incubated for 1 h with pifithrin α (Pthα), a specific inhibitor of p53 and thereafter non-exposed or exposed to 100 µM TMZ or 50 µM ACNU (left panel). LN229-Super and LN229-p53sh cells were non-exposed or exposed to 100 µM TMZ or 50 µM ACNU (right panel). Sensitivity was measured via MTT assay. **C.** Transcriptional induction of *fasR, ddb2* and *xpc* was analyzed using specific primers or as loading control, *gapdh* specific primers. **D.** LN-229 cells were exposed to 100 µM TMZ or 50 µM ACNU for the indicated times. Whole cell extracts were isolated, subjected to western blot analysis and induction of fasR, DDB2 and XPC protein was analyzed. Detection of ß-Actin was used as loading control.

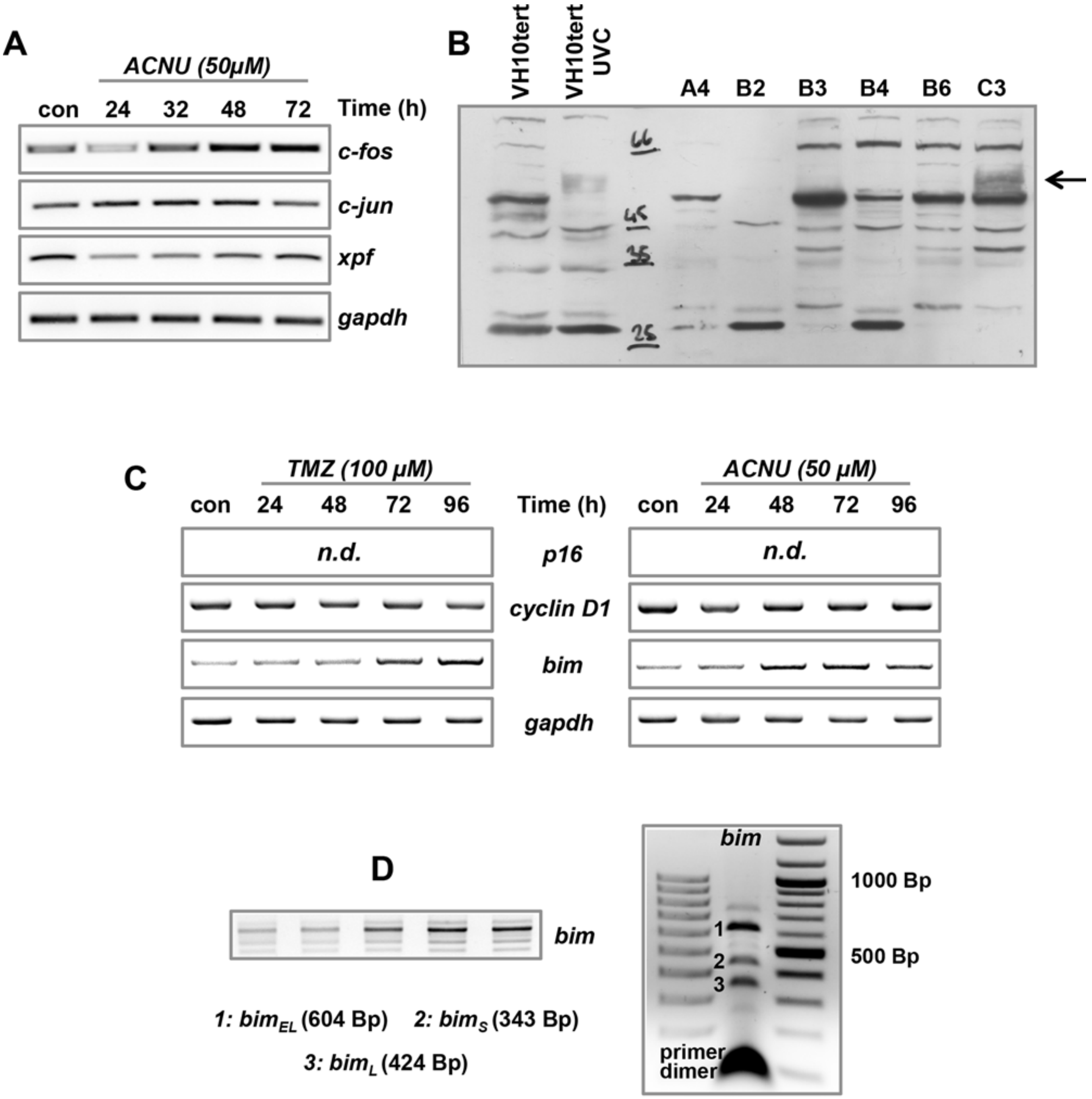

Supplementary Figure S2: Expression of *c-jun*, *c-fos* and *xpf* following ACNU exposure; c-Fos expression in different LN-229 cell clones and expression of *bim following* TMZ/ACNU. A. LN-229 cells were exposed to 100 µM TMZ or 50 µM ACNU for the indicated times. Transcriptional induction of *c-fos, c-jun* and *xpf* was analyzed using specific primers or as loading control, *gapdh* specific primers. **B.** Stable over-expression of c-Fos in various LN-229 cell-clones was detected by westernblot analysis. The specific band is indicated by an arrow. **C.** LN-229 cells were exposed to 100 µM TMZ or 50 µM ACNU for the indicated times. Transcriptional induction of *p16, cyclinD1* and *bim* was analyzed using specific primers or as loading control, *gapdh* specific primers. **D.** To identify the *bim* splice variant induced, the PCR was repeated using an increased cycle number (+3) and cDNA from TMZ exposed LN-229 cells (left figure). Moreover, the PCR reaction was repeated using an increased cycle number (+6) using cDNA from LN-229 cells 96 h upon TMZ exposure. Comparison with two different DNA markers was used to identify the three pro-apoptotic isoforms (bim_{EL} (604 Bp), bim_{L} (424 Bp), and bim_{S} (343 Bp) (right figure).

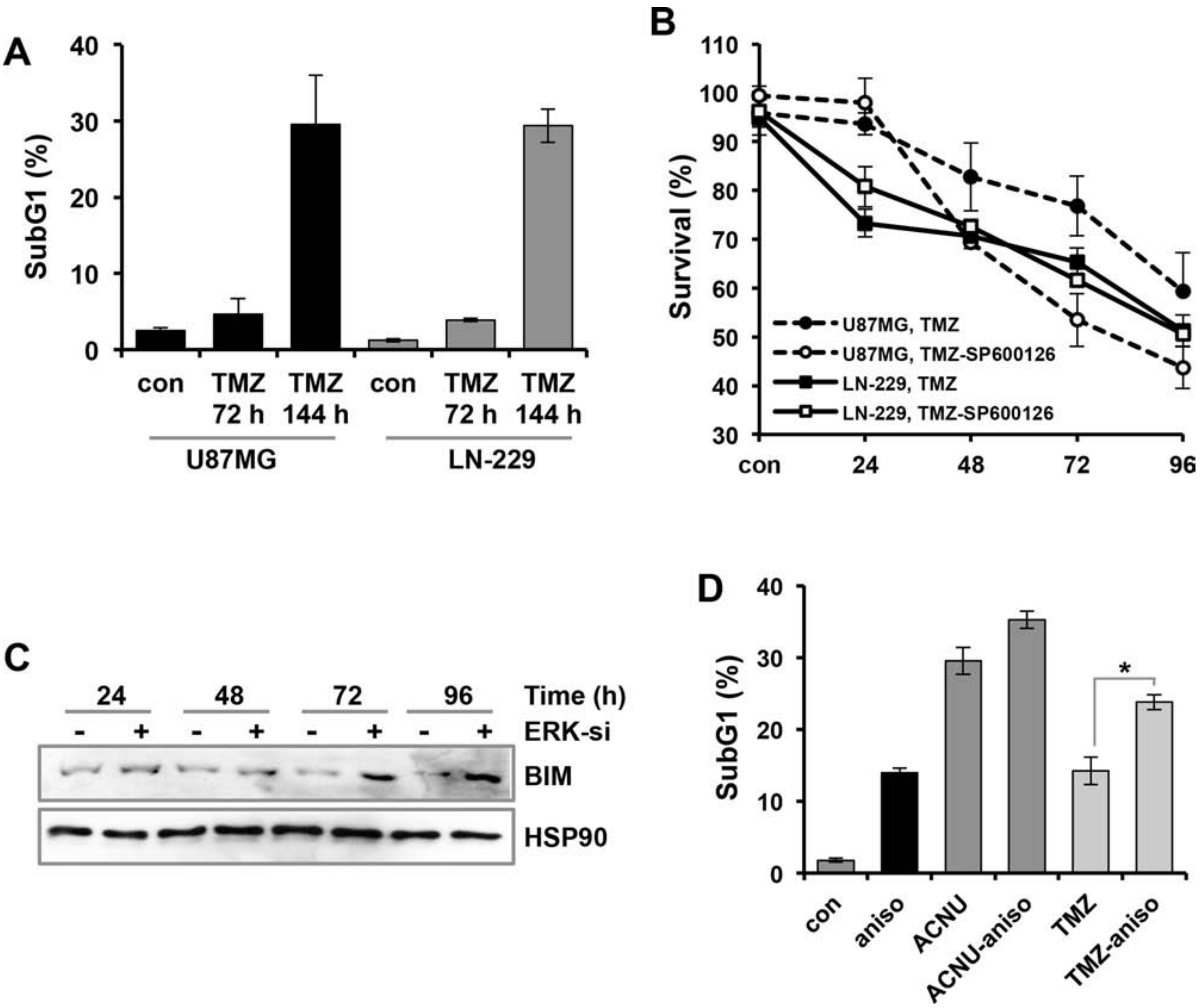

Supplementary Figure S3: TMZ-induced toxicity and metabolic competence in LN-229 and U87MG cells. A. LN-229 and U87MG cells were non-exposed or exposed to 100 µM TMZ for 72 or 144 h. The Sub-G1 fraction was determined by flow cytometry. **B.** LN-229 cells were pre-incubated for 1 h with a specific inhibitor for JNK1/2/3 (SP600125) and thereafter non-exposed or exposed to 100 µM TMZ for different time points. Metabolic competence was detected via MTT assay. **C.** LN-229 cells were transfected with 50 nM siRNA against ERK1/2 or a non-silencing RNA (ns). Expression of BIM was analyzed 24 - 96 h after transfection using specific antibodies. Detection of Hsp90 was used as loading control. **D.** LN-229 cells were treated or not treated with 0,1 µM anisomycin, 100 µM TMZ or 50 µM ACNU or combined treatment. Apoptosis was measured 96 h upon exposure *via* measurement of the subG1 fraction (right panel).

Supplementary Table S1: Primer sequences used in the study (5′ to 3′)

Gene	Primer sequence
gapdh-up	GAA GGT GAA GGT CGG AGT
gapdh-low	GAA GAT GGT GAT GGG ATT TC
fasR-up	AAG GGA TTG GAA TTG AGG AAG ACTG
fasR-low	GTG GAA TTG GCA AAA GAA GAA GACA
p21-up	GTG GGG GCA TCA TCA AAA ACT T
p21-low	CTC CCC CAT CAT ATA CCC CTA ACA
c-fos-up	GCA ATG AGC CTT CCT CTG A
c-fos-low	TCC AGC ACC AGG TTA ATT CC
c-jun-up	CGC GCC CTG GCC GAA CTG C
c-jun-low	CCT CTC CGC CTT GAT CCG CTC CTG
fasL-up	ATA TGT CAA CGT ATC TGA GCT CTC TCT
fasL-low	GAG TTC TAT GTT CTT CCG TCA TAT TCC
xpf-up	TCC CGT GCT TCT GAT TGA GTT TGA
xpf-low	AGG GCT GCT AAT TCT GCG ATG TTC
ddb2-up	GGG GCT CCA GCA GTC CTT TTT
ddb2-low	GGG CCA CAT GCG TCA CTT TCT TTT
xpc-up	CTT TGA TTT CCA TGG CGG CTA CTC
xpc-low	GCT GCT GCT TTC TTT TCC CTT TTG
bim-up	CCA AAT GGC AAA GCA ACC TTC TG
bim-low	CTG TCA ATG CAT TCT CCA CAC C
ccnd1-up	CAT CTA CAC CGA CAA CTC CAT CC
ccnd1-low	GAG GCG GTA GTA GGA CAG GAA GT
p16-up	CAA CGC ACC GAA TAG TTA CG
p16-low	GCA TGG TTA CTG CCT CTG GT

PCR program used: 1.5 min 94°C, [(denaturation: 45 sec, 94°C; annealing: 1 min 58–60°C; elongation: 1 min, 72°C) 21–30 cycles], 10 min 72°C.
